# Association between regular participation in sports and leisure time behaviors in Brazilian adolescents: A cross-sectional study

**DOI:** 10.1186/1471-2458-8-329

**Published:** 2008-09-23

**Authors:** Rômulo Araújo Fernandes, Ismael Forte Freitas Júnior, Jefferson Rosa Cardoso, Enio Ricardo Vaz Ronque, Mathias Roberto Loch, Arli Ramos de Oliveira

**Affiliations:** 1 Student of the MSc. in Physical Education Program, Universidade Estadual de Londrina, Rodovia Celso Garcia Cid km 380, 86051-990 Londrina, PR, Brazil; 2 Department of Physical Education, Universidade Estadual Paulista, Rua Roberto Simonsen 305, 19060-900 Presidente Prudente, SP, Brazil; 3 MSc. in Physical Education Program, Universidade Estadual de Londrina, Rodovia Celso Garcia Cid km 380, 86051-990 Londrina, PR, Brazil

## Abstract

**Background:**

The belief that adolescents engaged in sports increase their overall physical activity level while simultaneously decreasing physical inactivity has been the foundation of many intervention programs in developing countries. The aim of this study was to analyze the association between regular participation in sports and both active behaviors and TV viewing during leisure time.

**Methods:**

A total of 1752 Brazilian adolescents (812 = male and 940 = female) participated in this study. Regular participation in sports, as well as active behaviors (exemplified by walking or cycling) and TV viewing during leisure time were assessed by means of a questionnaire. The chi-square test analyzed the association between sports practice and leisure time behaviors, and the Poisson regression with robust variance indicated the magnitude of these associations.

**Results:**

The prevalence of regular participation in sports was 14.8% (95% confidence interval 13.2% to 16.5%). After adjustment for all confounders, participation in sports was associated with, at the highest frequency, cycling (PR = 2.55 [1.80–3.60]) and walking (PR = 2.69 [1.98–3.64]) during leisure time. However, there was not an association between the participation in sports and frequency of TV viewing (PR = 1.28 [0.81–2.02]).

**Conclusion:**

This study presented data indicating that the regular participation in sports is positively associated with a higher frequency of physically active behaviors during leisure time. However, the results did not support the hypothesis that the engagement in sports necessarily decreases leisure time spent in TV viewing.

## Background

In recent decades, physical inactivity has become one of the most pressing public health problems among both developing and developed countries [1,2]. However, in developing countries, such as Brazil, data about this issue are scarce. The concern over physical inactivity is justified because it is a primary factor in the development of many chronic diseases, which not only lead to productivity loss, but also increase the public health care burden [2]. On the other hand, regular physical activity (PA) is associated with lifelong beneficial health effects [3-6].

Physically active children and adolescents who engage in sports are more likely to be physically active during early adolescence and adulthood, respectively [7,8]. Thus, in developing countries, both children and adolescents have been the special focus of sports-promoting public health strategies. These strategies have normally targeted to increase the physical activity level (PAL) of these populations, assuming that, thereby, the level of physical inactivity would be decreased.

It is usually accepted that media-based sedentary behaviors such as television viewing (TV viewing), surfing the internet, and playing video games consume time that might otherwise be spent in physical activity. Furthermore, these sedentary behaviors may promote snacking between meals [9], which is linked to obesity and insulin resistance [4,10]. Moreover, Hesketh et al. [11] indicated that, among Australian children, sedentary behaviors increased with age when tracked over time.

Among adolescents, TV viewing is considered an important indicator of sedentary behaviors because in both developed and developing countries the number of televisions per person increased positively with the rate of overweight [12]. Nevertheless, recent studies have indicated that PA and TV viewing are dissociated [4,13] and differently associated with adiposity and metabolic risks [4].

This possible dissociation between PA and TV viewing has obvious relevance for public health, because, if it were actually the case, the design of health-promotion strategies should be drastically altered. Such strategies, then, would not only have to increase PA during childhood and adolescence, but also develop separate mechanisms for decreasing time spent in TV viewing. Hence, due to the scarcity of data regarding the existence and degree of this dissociation, it is necessary to discover the interrelation of these variables, thereby facilitating the creation of better informed intervention programs.

Thus, the primary hypothesis of this study was that higher participation in sports would be positively associated with an elevated frequency of other active behaviors during leisure time. The secondary hypothesis, then, was that the engagement in sports would be also be positively associated with a decreased frequency of TV viewing.

The presented study analyzed the relationship between regular participation in sports and both TV viewing and active behaviors (represented by walking and cycling) during leisure time in a sample composed of Brazilian adolescents.

## Methods

### Sample

This cross-sectional study was carried out in the city of Presidente Prudente (HDI = 0.846), Southeastern Brazil, from July to October 2007. The city of Presidente Prudente is located in the western part of the state of São Paulo and has approximately 200,000 inhabitants, of whom ~37,000 are students ranging in age from 11 to 17 years old, distributed in 118 primary and secondary public (~70% of all students) and private (~30% of all students) schools.

The sample size of 1495 subjects was estimated by means of an assumed PA prevalence of 41.8% [14] (error of 2.5%), with a power of 80%, and an alpha error of 5%. From the thirty-six middle and high schools which serve the determined age range, six were selected randomly conforming to the proportion of students in public (four schools: *n *= 1290 [73.6%]) and private schools (two schools: *n *= 462 [26.4%]). In each school, all of the students were invited to participate and received an informed written consent form, which was filled out by both the parents and the students before participating in the survey. The study was approved by the Ethics Committee on Human Experimentation of the Sao Paulo State University at Presidente Prudente.

### Measurements

The data were collected during physical education class. The participants self-reported information via two questionnaires: PA and family socio-economic status (F-SES). Participation in sports, as well as the other leisure time behaviors, were assessed by the questionnaire developed by Baecke et al. [15]. A trained researcher administered the questionnaire. The adolescent was considered regularly engaged in sports if he participated any organized sport of moderate to vigorous intensity more than 4 hours per week in the three months prior to the study. Physical education classes were not included in this study because these were carried out infrequently (generally only once per week) and involved physical activities of low intensity.

Data regarding leisure time activities (walking, cycling, and TV viewing) were also collected. The frequency (never; seldom; sometimes; often; very often) of these behaviors (active = walking/cycling; TV viewing) were collected and grouped into three categories: Low frequency (L-Frequency = never and seldom), Mid frequency (M-Frequency = sometimes and often), and High frequency (H-Frequency = very often).

Potential confounder variables such as age, gender, level of parent education, and F-SES (according to the Brazilian Criterion for Economic Classification proposed by ANEP [16]) were also collected. The questionnaire for F-SES covered the parent's schooling, presence/absence and number of domestic appliances, vehicles, and rooms in the adolescent's home, and the family was classified into categories from A (wealthiest) to E (poorest). Parents were also invited to participate by letter. At home, they filled out a questionnaire in which they reported their own schooling. To analyze the consistency of the adolescents' and parents' reported data, one hundred and seventy adolescents and thirty parents were randomly selected and invited to participate in an interview at the school, where a researcher re-administered the questionnaire. The data's agreement level (Kappa statistic [*k*]) between the two measures was high for both parents (*k *= 1.0) and adolescents (*k *= 0.85 [PA] and *k *= 0.87 [F-SES]).

### Statistical procedures

Initially, the chi-squared test (χ^2^) was used to evaluate the crude association between regular participation in sports and all leisure time behaviors. After the χ^2 ^use, the Poisson regression with robust variance, represented by the prevalence ratio (PR) and 95% confidence interval (95%CI), was applied to indicate the possible effects of confounders (age, gender, parent's schooling, and F-SES). For the categorical variables, the agreement level was analyzed by the Kappa statistic. STATA 8.0 (Stata Inc., College Station, TX, USA) was used for all data management and all analyses were performed accounting for the sample design (*svy *set of commands available in STATA 8.0). The significance was set at 5%.

## Results

In the six schools, from 2200 eligible students, 1752 participated in the study (response rate = 79.6%). Among the 448 students who refused participate, 198 were male and 205 female adolescents (44.2% and 55.8%, respectively; p = 0.014) and the mean age of this group was 13.1 (SD 1.5). Approximately 15% (n = 67) of the students who refused participate were enrolled in private schools. Among the 1752 adolescents analyzed, the response rate to both questionnaires was high (PA: 98.8%; F-SES: 100%).

In Table 1 the demographic and socio-economic variables of the analyzed sample are presented. There were more female than male adolescents (*p *= 0.002). Approximately 25% of the parents completed more than fourteen years of schooling and almost 30% of the sample was grouped into the highest F-SES. The prevalence of adolescents regularly engaged in sports was only 14.8% (95% confidence interval 13.2% to 16.5%) and there was an association with gender (male: 21.2% and female: 9.4%; *p *= 0.001).

**Table 1:** General information of the sample (Brazil, *n *= 1752).

Variables	n	(%)
Gender		
Male	812	(46.3)
Female	940	(53.7)
Age (years)		
11–14	1098	(62.7)
15–17	654	(37.3)
Paternal schooling		
≥ 15 years	410	(23.5)
<15 years	1342	(76.5)
Maternal schooling		
≥ 15 years	400	(22.8)
<15 years	1352	(77.2)
F-SES		
A (wealthiest)	496	(28.3)
B	862	(49.2)
C – D – E (poorest)	394	(22.5)
Participation in sports		
Yes	260	(14.8)
No	1492	(85.2)

F-SES = family socio-economic status.

The highest rate of L-frequency response (47.9%) was observed in the behavior 'walking' ('TV viewing': 8.6% and 'cycling': 46.1%), and the highest rate of H-frequency response (36.8%) was observed in the behavior 'TV viewing' (walking: 9.2% and cycling: 16.7%).

Of all analyzed leisure time behaviors, only the frequency of TV viewing was not associated with the frequency of walking (*p *= 0.142), however, it had an association with the frequency of cycling (*p *= 0.001). There was also an association among all the other active leisure time behaviors (data not shown).

Table 2 shows the association among leisure time behaviors and any potential confounder (gender, age and F-SES). A higher frequency of TV viewing was negatively associated with gender (female), higher age, higher F-SES, and higher parent schooling. A higher frequency of walking was positively associated only with lower maternal schooling, and the frequency of cycling was significantly associated with all potential confounders.

**Table 2:** Association among behaviors during leisure time and potential confounders (Brazil, *n *= 1752)

Leisure Time	Gender (%)		Age (%)		F-SES (%)	
	Male	Female	11–14	15–17	Wealthiest	Poorest

TV viewing						
L- Frequency	11.2	6.4	5.6	13.7	7.4	11.7
M- Frequency	59.9	49.9	49.6	62.9	52.1	63
H- Frequency	28.9	43.6	44.8	23.5	41.4	25.3
	p = 0.001		p = 0.001		p = 0.001	
Walk						
L- Frequency	49.4	46.5	49.2	45.7	47.8	48
M- Frequency	41.6	44	41.2	45.7	42.2	44.7
H- Frequency	9	9.5	9.6	8.6	10	7.3
	p = 0.282		p = 0.446		p = 0.411	
Cycle						
L- Frequency	32.4	57.6	39	57.6	44.8	49
M- Frequency	41.2	33.8	39.9	32.8	37.4	36.7
H- Frequency	26.4	8.6	21.1	9.6	17.8	14.3
	p = 0.001		p = 0.001		p = 0.050	

F-SES = family socio-economic status; L-Frequency = low frequency; M-Frequency = middle frequency; H-Frequency = high frequency.

Table 3 shows the association between regular participation in sports and the frequency of the three analyzed behaviors. In univariate model, the regular participation in sports was positively associated with a higher frequency of walking and cycling during leisure time. However, the frequency of TV viewing was not associated with participation in sports.

**Table 3:** Prevalence ratio and 95% confidence interval for behavior factors associated to sports practice (Brazil, *n *= 1752).

		Regular Participation in Sports	
Leisure Time	Sports (yes)	Crude	Adjusted*
	n (%)	PR [95%CI]	PR [95%CI]

TV viewing			
L-Frequency	27 (20.1)	1.00	1.00
M-Frequency	142 (17.3)	1.00 [0.64–1.58]	1.13 [0.72–1.76]
H-Frequency	95 (16.9)	0.98 [0.62–1.56]	1.28 [0.81–2.02]
		p = 0.989	p = 0.577
Walk			
L-Frequency	93 (12.1)	1.00	1.00
M-Frequency	119 (18.3)	1.33 [1.00–1.76]	1.32 [1.00–1.74]
H-Frequency	58 (50.9)	2.79 [2.05–3.79]	2.69 [1.98–3.64]
		p = 0.001	p = 0.001
Cycle			
L-Frequency	78 (10.5)	1.00	1.00
M-Frequency	97 (17.2)	1.71 [1.24–2.34]	1.56 [1.13–2.16]
H-Frequency	87 (41.1)	3.03 [2.22–4.13]	2.55 [1.80–3.60]
		p = 0.001	p = 0.001

* = adjusted for gender, age, family socio-economic status and parent's schooling; L-Frequency = low frequency; M-Frequency = middle frequency; H-Frequency = high frequency; PR = prevalence ratio; 95%CI = confidence interval of 95%.

In the multivariate model, after the adjustment for confounders, the two associations remained statistically significant. Again, there was no association between TV viewing and regular participation in sports.

## Discussion

This cross-sectional study enrolled adolescents of both genders and indicated that, even after adjustment for important confounders associated with PA, regular participation in sports was associated with active leisure time behaviors, but not with sedentary behaviors.

The presented study has several strengths which provide adequate external validity for the results found, such as its large sample size and the control of the design effect in the analyses. Additionally, the high agreement level for collected data is a good indicator of internal validity. However, several limitations also should be recognized. Although the results found were similar to other publications which measured the total PAL [4,13,17], the measurement of a fraction of the PA (participation in sports during leisure time) instead of the total amount should be considered as a limitation which could affect the observed high rate of inactivity. Another considered limitation of this study is the sample size calculation, which assumed a prevalence of 41.8% when the observed overall rate of the outcome was approximately 15%. This discrepancy could have implications on the power of the study and, therefore, in the detection of the differences. However, a new sample size was estimated within the same parameters (error of 2.5%, power of 80% and significance of 5%) and including the discovered rate of regular participation in sports, 14.8% (n = 775). A sub-sample was randomly selected and the statistical analyses were again carried out (data not shown). Even with slight differences in the PR values, there was similarity between all sub-sample analyses and the overall sample results in both crude and adjusted models; this fact minimizes the likelihood of bias in the presented results.

Although adopting different cut-offs, the regular engagement in sports observed in these adolescents was low (14.8%) in comparison with those analyzed by Baumert et al. [18] who identified a rate of American adolescents engaged in sports of 59%. These findings are in agreement with the high prevalence of sedentary lifestyle observed among Brazilian adolescents [14], and indicated that in this population effective strategies for physical activity promotion are needed.

Furthermore, there was an associated between male gender and higher engagement in sports, which concurs with other studies performed in both more and less developed settings [4,7]. Recently, Gonçalves et al. [19] reported that Brazilian male adolescents had more social and family support to engage in physical activities than female adolescents. Furthermore, in Brazil the prevalence of perceived personal barriers to engagement in active leisure-time behaviors is more frequent in the female gender [20].

The relationship observed between the regular participation in sports and all active behaviors confirms the first hypothesis of this study, since the positive and significant effect of the practice of sports on two active behaviors examined remained statistically significant after adjustment for important confounders associated with PA, such as gender and family socio-economic status. These findings are consistent with those of Baumert et al. [18] in which adolescents not engaged in sports less often reported getting 30 minutes of nonstop exercise three times a week than did those engaged in sports.

Likewise, in a previous publication, Larson et al. [21], who analyzed the association between the participation in sports and unhealthy behaviors, indicated that American adolescents who smoke cigarettes might be less likely to participate in ≥1 team sports. Among Brazilian female adolescents, the participation in sports was a protective factor associated with a lower likelihood of pregnancy and early onset of sexual intercourse [22]. These data indicate that the regular participation in sports during adolescence is associated with other healthy lifestyle attitudes; thereby, its relevance for physical health is confirmed, which justifies its continued presence in developing countries' public health programs.

Marshall et al. [23] in a meta-analysis indicated that, although too small, there is a statistically significant relationship between TV viewing and body fatness. Further, in a recent review, Bryant et al. [9] presented four possible mechanisms that might explain this relationship (impact of TV viewing on weight gain), and one of these mechanisms was that television displaces time that would otherwise be used for physical activity.

In this present study, the results did not indicate that the regular participation in sports decreased TV viewing during leisure time, and for this reason, the secondary hypothesis of the present study was rejected.

Although several important differences in the methods used in this study, such as the cross-sectional design and PA questionnaire use, must be considered for comparison of our results with previous data, this dissociation agrees with other studies from more developed nations. Ekelund et al. [4] among European children and adolescents in cross-sectional analysis, observed that the time spent on TV viewing and free time PA measured by accelerometer were not correlated (*r *= 0.01). Burke et al. [17], among Australian adolescents, also observed no relation between the time spent in PA during weekdays and TV viewing. Furthermore, Taveras et al. [13] in a longitudinal study (4 years of follow-up), indicated that changes in TV viewing were not associated with changes in leisure time activities of higher intensities.

Thus, this lack of association seems to occur in both developed and developing countries. Future national surveys, principally in the context of developing nations, should analyze physical activity and inactivity as independent entities and not as the same variable anymore. Besides this, the implications of these results for public health policies are that the newer PA-promotion strategies should not only increase PAL, but also create separate strategies for decreasing the time spent on sedentary behaviors. Another implication is that in clinical practice, health professionals must take sedentary behaviors into account as important risk factors which are not beneficially influenced by PA and, hence, must create tools for combating these behaviors among pediatrics populations.

## Conclusion

In conclusion, this study presented data indicating that the regular participation in sports during adolescence is positively associated with higher frequencies of other active behaviors during leisure time. Furthermore, there was dissociation between regular sports practice and frequency of TV viewing, indicating that the results did not support the hypothesis that engagement in sports decreases the time spent on TV viewing during leisure time.

## Abbreviations

PA: physical activity; PAL: physical activity level; L-Frequency: low frequency; M-Frequency: mid frequency; H-Frequency: high frequency; PR: prevalence ratio; 95% CI: confidence interval of 95%.

## Competing interests

The authors declare that they have no competing interests.

## Authors' contributions

RAF was the principal researcher responsible for the collection, analysis and interpretation of data, as well as for drafting the manuscript. JRC was involved in analysis and interpretation of data and also in critical revision of the paper. IFFJ,  ERVR, MRL and AROwere involved in revising the manuscript critically for important intellectual content.

## Pre-publication history

The pre-publication history for this paper can be accessed here:


